# Monofrequency electrical impedance mammography (EIM) diagnostic system in breast cancer screening

**DOI:** 10.1186/s12885-020-07283-5

**Published:** 2020-09-14

**Authors:** Blanca Murillo-Ortiz, Abraham Hernández-Ramírez, Talia Rivera-Villanueva, David Suárez-García, Mario Murguía-Pérez, Sandra Martínez-Garza, Allyson Rodríguez-Penin, Rosario Romero-Coripuna, Xiomara Midory López-Partida

**Affiliations:** 1 Unidad de Investigación en Epidemiología Clínica, Unidad Médica de Alta Especialidad No. 1 Bajío, Instituto Mexicano del Seguro Social, B. López Mateos Esq. Insurgentes S/N, Colonia: Los Paraísos, CP 37320 Leon, Guanajuato Mexico; 2 Servicio de Radiología e Imagen Unidad Médica de Alta Especialidad No. 48, Instituto Mexicano del Seguro Social, Leon, Guanajuato Mexico; 3 Departamento de Radiología, Hospital General de Zona No. 58, Delegación Guanajuato, Instituto Mexicano del Seguro Social, Leon, Guanajuato Mexico; 4 Departamento de Oncología, Unidad Médica de Alta Especialidad No. 1 Bajío, Leon, Guanajuato Mexico; 5 Departamento de Patología, Unidad Médica de Alta Especialidad No. 1 Bajío, Instituto Mexicano del Seguro Social, Leon, Guanajuato Mexico; 6 Unidad de Medicina Familiar No. 51, Delegación Guanajuato, Instituto Mexicano del Seguro Social, Leon, Guanajuato Mexico

**Keywords:** Breast cancer, Screening, Frequency electrical impedance

## Abstract

**Background:**

Some evidence has shown that malignant breast tumours have lower electrical impedance than surrounding normal tissues. Electrical impedance could be used as an indicator for breast cancer detection. The purpose of our study was to analyse the sensitivity and specificity of electrical impedance mammography (EIM) and its implementation for the differential diagnosis of pathological lesions of the breast, either alone or in combination with mammography/ultrasound, in 1200 women between 25 and 70 years old.

**Methods:**

This study is a prospective, cross-sectional epidemiological observational study of serial screening. The women were invited to participate and signed a consent letter. Impedance imaging of the mammary gland was evaluated with the computerized mammography equipment of MEIK electroimpedance v.5.6. (0.5 mA, 50 kHz), developed and manufactured by PKF SIM-Technika®. The successful identification of breast cancer along with the sensitivity, specificity, and positive and negative predictive values of EIM were determined as follows: % sensitivity; % specificity; % positive predictive value (PPV); and % negative predictive value (NPV).

**Results:**

EIM had a sensitivity of 85% and a specificity of 96%; the positive predictive value was 12%, and the negative predictive value was 99%. Seven cases were biopsy confirmed cancers. Significant correlations between the electrical conductivity index and body mass index (BMI) (*p* = 0.04) and patient age were observed (*p* = 0.01). We also observed that the average conductivity distribution increased according to age group (*p* = 0.001). We used the chi-squared test to assess the interactions between percent density and BMI (normal < 25 kg/m2 (*n* = 310), overweight 25–29.9 kg/m2 (*n* = 418) and obese ≥30 (*n* = 437)) (*p* <  0.05). The patients with a diagnosis of mammary carcinoma had a BMI of 35.51 kg/m2.

**Conclusions:**

Our results demonstrate that the use of monofrequency electrical impedance mammography (EIM) in the detection of breast cancer had a sensitivity and specificity of 85 and 96%, respectively. These findings may support future research in the early detection of breast cancer. EIM is a non-radiation method that may also be used as a screening method for young women with dense breasts and a high risk of developing breast cancer.

## Background

Electrical impedance mammography is a relatively new method for the diagnosis of mammary diseases [1]. In a short period of time, diagnostic criteria have been developed for the early detection of breast cancer by means of electrical impedance imaging [2]. It is a noninvasive diagnostic technique based on the electrical storage potential difference between normal and pathologically altered tissues that allows image differences in the conductivity and permittivity of inferred tissue from electrical measurements of the body surface. Mammography by electrical impedance belongs to the class of 3D tomography systems [3].

Raneta et al. Reported that electroimpedance mammography showed a sensitivity of 87% with a specificity of 85%. The use of electroimpedance mammography in addition to mammography and ultrasound (MMG/USG) can improve the sensitivity of these methods and increase the rate of the early detection of breast cancer with minimal economic costs and highly qualified staff time expenditures [3].

The electrical impedance mammography (EIM) monofrequency equipment does not emit radiation, it was created by Modern Impedance Medical Equipment (MEIK), recently used in high-risk groups, as well as the effective follow-up of cancer treatment, with great sensitivity and specificity [4].

Its efficiency was estimated to be 87.39%. Of 75 patients with breast cancer, 96% were found to have a degree III risk of disease progression; 4% had a degree II risk of disease progression. Additional examination was recommended for these patients. Considering that EIM works without any type of ionizing radiation, it can be recommended to be used in pregnant patients, hospitalized patients, and ambulatory, family medicine and obstetrics and gynaecology units for the screening of women under 40 years of age [5].

The electric conductivity index (IC) obtained from electrical impedance scanning is a quantitative variable that characterizes the mammary gland structure. A low index is typical of a gland containing a large number of cell elements and therefore a high ion concentration [6].

Thus, the mammary gland structure can be assessed from the perspective of electrical impedance mammography with regard to the electric conductivity index. The mammary gland structure determines its density, so that the different ranges of electrical conductivity correspond to different degrees of mammary density [7, 8]. The assessment is performed in line with the American College of Radiology (ACR) guidelines [9].

Wang K et al. Showed that there are significant differences in the properties of electrical impedance between cancerous tissue and healthy tissue. The impedance of benign tumours is smaller and is at the same level as that of the mammary glandular tissue. The different growth patterns of mammary lesions determines the different electrical impedance characteristics in the EIS results [10].

The structural types of the breast have been defined according to the correlation between the ductal component and the fat lobes, and the breast can have a variable appearance in the tomogram. This is why the mammographic scheme of electrical impedance depends on the type of mammary structure; the structure of the mammary gland is studied from the perspective of mammography by electrical impedance according to the degrees of mammary density of the ACR classification [11]. A volumetric lesion is an affectation that is detected in several planes of exploration [12]. The analysis of the image involves the evaluation of the shape of the lesion: the contour, the internal electrical structure and the changes in the surrounding tissues were scored between 0 and 2 for each alteration or pathological finding [13]. The conduction of electric current through a tissue can be affected by changes in the tissue structure and composition due to, for example, cell proliferation and tumour growth [14]. The sum of the scores is stratified into a scale impedance score of 5 degrees BI-EIM, which is in great agreement with the BI-RADS classification system [15]. The BI-EIM 4 and 5 scores are considered positive and are referred to biopsy [16]. The use of a numerical score for the evaluation of the volumetric lesions by the electroimpedance of the breast allows a comparison of this information with the BI-RADS categories.

Cancer cells exhibit altered local dielectric properties compared to normal cells, measurable as different electrical conductance and capacitance by electrical impedance scanning [17]. The divergence of the distribution form of the histograms should be evaluated. During the development of the oncological process, the general and local electrical conductivity naturally tends to change. The distortion of the mammographic scheme can be observed from the onset of the disease. Comparative conductivity is the alteration of the electrical conductivity of one breast with respect to the other.

The EIM point scale allows the standardization of the description of volumetric lesions when performing an electrical impedance mammography examination (Table 1), as well as the use of the patient monitoring algorithm developed by the specialists of the American College of Radiology (Table 2). Electrical conductivity rates have been considered useful data for clinicians to guide diagnosis and treatment decisions and can be used to evaluate breast tissue as a reliable tool for both individual and complementary use [18].

**Table 1:** Diagnostic criteria for the differentiation of volumetric lesions in electroimpedance mammography

Diagnostic criteria	Electrical impedance mammography points (EIM)
**Shape**	
**Round, oval**	**1**
**Lobular, irregular**	**2**
**Contour**	
**No**	**0**
**Sharp**	**1**
**Hyperimpedance, indistinct**	**2**
**Surrounding tissues**	
**Preserved**	**0**
**Structure alteration/displacement**	**1**
**Thickening/extrusion/retraction**	**2**
**Internal electrical structure**	
**Hyperimpedance**	**0**
**Isoimpedance**	**1**
**Hypoimpedance**	**2**
**Animpedance**	**3**
**Comparative electrical conductivity**	
**Divergence between the histograms < 20%**	**0**
**Divergence between the histograms 20–30%**	**1**
**Divergence between the histograms 30–40%**	**2**
**Divergence between the histograms > 40%**	**3**

**Table 2:** EIM scale and ACR BIRADS

EIM	ACR
**Common scale**	**BI-RADS Categories**
**No score**	
**0–1**	**BI RADS 0 Poor image**
**2–3**	**BI-RADS 2 Benign tumours-routine mammography**
**4**	**BI-RADS 3 Probably benign findings**
**5–7**	**BI-RADS 4 Suspicious abnormality-biopsy**
**> 8**	**BI-RADS 5 Highly suggestive of malignancy-treatment/biopsy.**

To determine the sensitivity and specificity of mammography by monofrequency electroimpedance, we screened 1200 women from 25 to 70 years of age who underwent EIM examinations as part of this study.

## Methods

### Study design and populations

This is a prospective, observational epidemiological study of a cross-section screening of breast cancer in a series of Medical Units of High Specialty, Number 1, Guanajuato Delegation of the Mexican Institute of Social Security. The women were invited to participate and signed a consent letter. This protocol was approved by the bioethics committee (R-2017-785-108). Written informed consent was obtained from each volunteer.

Four groups were formed: Group 1 = 25 to 35 years, Group 2 = 36 to 45 years, Group 3 = 46 to 55 years and Group 4 = 56 to 70 years. Impedance imaging of the mammary gland was evaluated with the computerized mammography equipment of MEIK electroimpedance v.5.6 (0.5 mA, 50 kHz), developed and manufactured by PKF SIM-Technika®.

All women aged ≥40 years were subjected to screening with conventional mammography (asymptomatic) and complementary ultrasound. Doppler ultrasound was performed in those < 40 years old with BIRADS 3 to 5. In addition to collecting data on the results of the EIM examination and other breast examinations, menopausal status and exogenous hormone use were also recorded.

### Monofrequency electrical impedance mammography (EIM)

EIM was performed while the patient was at rest in the dorsal decubitus position, and the electrodes were placed. For the recording of conductivity, two electrodes were placed on the arm, first on the right arm to study the left breast and then on the left arm to study the right breast. The interpretation of the study consists of the analysis of images [4].

**Fig. 1:**
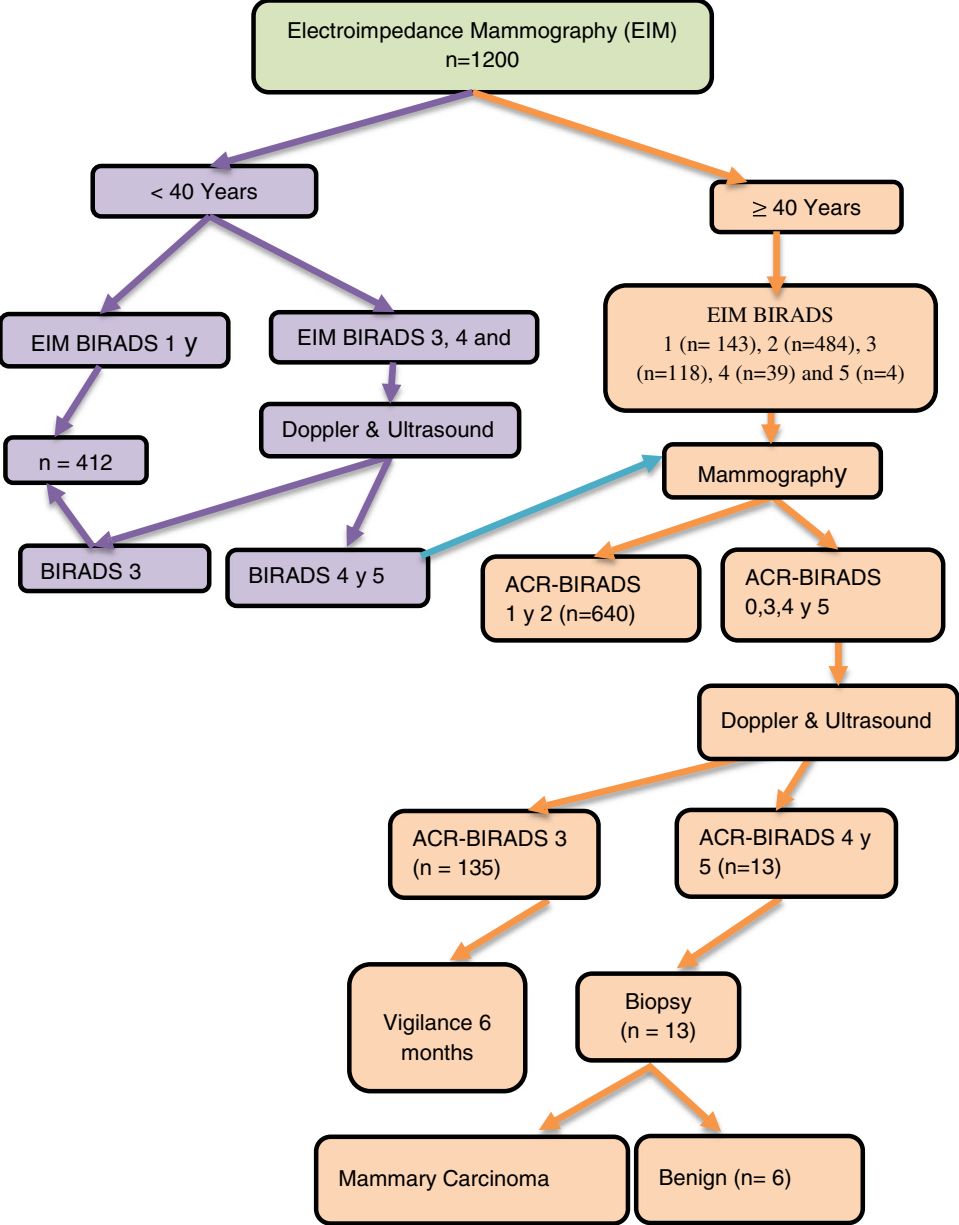
Flow Chart

A diagnostic table was made to regularize the description of volumetric lesions. The Table 1 show contains assessment parameters, each being given a certain number of points, electrical impedance mammography points (EIM) (Table 1). Using the numerical score for the assessment of volumetric lesions in electrical impedance mammography allows us to compare this information to ACR BI-RADS (Breast Imaging Reporting and Data System) (Table 2). To determine the sensitivity and specificity of the mammography study with electroimpedance, it was carried out at the beginning, and later, the concordance with mammography and ultrasound imaging studies was analysed. In Fig. 1 we can see the flow diagram, the final diagnosis was made by biopsy and histopathological study (Fig. 1).

### Mammary gland structure and density types

Percent density was measured with the EIM Classification to evaluate the associations between percent density, age and body mass index subgroups (normal/underweight, < 25 kg/m2 versus overweight/obese, ≥ 25 kg/m2). We used the Wald chi-squared test to assess the interactions between percent density and body mass index.

### Statistical analysis

The successful identification of breast cancer along with the sensitivity, specificity, and positive and negative predictive values of EIM were determined as follows: % sensitivity; % specificity; % positive predictive value (PPV); and % negative predictive value (NPV).

We analysed the following clinical factors: age, divided into four categories (25 to 35 years, 36 to 45 years, 46 to 55 years and 56 to 70 years); menopausal status, premenopausal versus postmenopausal (a woman was considered postmenopausal if she had gone 6 months without menstruating); exogenous hormone use (oral contraceptives, contraceptive implants, or intrauterine devices with hormones if premenopausal; hormone replacement therapy if postmenopausal); previous history of breast cancer, yes or no; family history of breast cancer (categories were no first-degree relatives with breast cancer, one first degree [sister/mother] relative with breast cancer, or two or more first-degree relatives with breast cancer); palpability of lesion (palpable mass present or not present); and breast tissue density.

The methods included the Breast Imaging Reporting and Data System (BI-RADS) numerical system. The distribution of mammary gland structure and density types from the perspective of EIM execution were in accordance with the ACR classification. For the purposes of this analysis, the women were classified into one of four categories: predominantly fat, fat with some fibroglandular tissue, heterogeneously dense, and extremely dense. All data are presented as the mean ± SE, with *P* values < 0.05 considered significant.

## Results

The study involved 1200 female participants. The patient characteristics are shown in Table 3. The patients had a median age of 47.58 ± 11.39 years (range 25–70 years).

**Table 3:** Demographic and Clinical Characteristics of the Women at the Time of Entry into the Study (*n* = 1200)

	Group 1 ***n*** = 196	Group 2 ***n*** = 319	Group 3 ***n*** = 393	Group 4 ***n*** = 292	p
Age (years)	29.82 ± 3.92	41.60 ± 2.66	50.22 ± 2.74	62.5 ± 4.75	P = 0.001
Electrical Conductivity Index	0.35 ± 0.11	0.41 ± 0.11	0.49 ± 0.12	0.54 ± 0.10	*P* = 0.001
BMI kg/m2	26.02 ± 5.78	28.94 ± 5.81	29.12 ± 5.70	29.37 ± 6.06	P = 0.001
% Fat	33.79 ± 7.67	37.41 ± 7.38	38.09 ± 6.76	38.40 ± 7.89	P = 0.001
Menopausal status Postmenopausal (%)	6.12	12.5	58.77	96.23	P = 0.001
Hormone use (%)	46.93	35.42	34.86	34.24	P = 0.001
Smoking (%)	17.85	11.91	9.41	7.87	P = 0.001
Alcoholism (%)	15.81	11.91	7.88	5.13	P = 0.001
Previous history of breast cancer (%)	0	0	0	0	ns
Family history One first-degree relative with breast cancer (%)	6.63	8.46	8.90	10.27	P = 0.001
Palpable lesion(%)	19.38	22.88	18.06	13.01	P = 0.001

The data are shown as the mean ± SD. The Mann-Whitney test was used to determine the differences

Four groups were formed, the first with 196 women between 25 and 35 years old, the second with 319 women between 36 and 45 years old, the third with 393 women between 46 and 55 years old and the fourth with 292 women between 56 and 70 years old. The body mass index (BMI) of these patients was 28.63 ± 5.94 kg/m2. The anthropometric distribution was as follows: percentage of body fat: 37.28 ± 7.52%, muscle: 41.77 ± 5.45%, water: 44.58 ± 16.67%, visceral fat: 8.21 ± 3.52%, and bone: 2.27 ± 0.82%. Normal weight (< 25 kg/m2) was observed in 310 (26.60%) women, and overweight (25–29.9 kg/m2) was observed in 418 (35.87%) women. A total of 294 (25.23%) patients had grade I, 96 (8.24%) had grade II, and 47 (4.03%) had grade III breast cancer.

### Electrical conductivity index

**Fig. 2:**
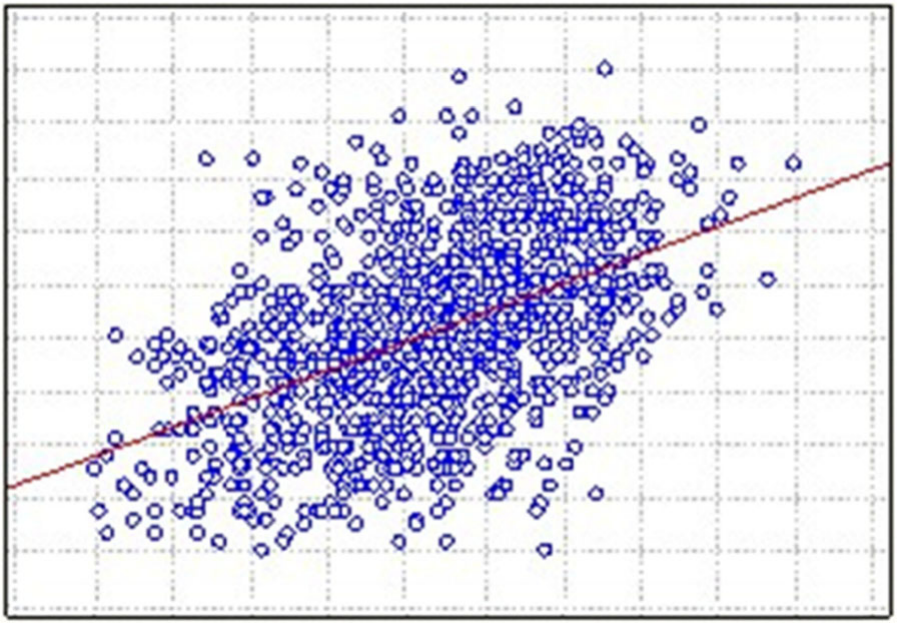
Correlation of electrical conductivity in the mammary tissue and age of the patients. *p* < 0.05 was considered significant

When analysing the electrical conductivity in the mammary tissue of the patients, it was observed that the average conductivity distribution increased according to the age group (r = 0.49, *p* = 0.001) (Fig. 2). Group 4, which corresponds to women aged 56 to 70 years, presented the highest conductivity, with a mean of 0.53 ± 0.11, and group 1, which corresponds to women aged 25 to 35 years, had the lowest conductivity, of 0.36 ± 0.11, with a statistically significant difference (*p* = 0.001).

The difference in the distribution of conductivity between the mammary glands of the total group was 10.15 ± 5.18; the conductivity in the left breast was 0.48 ± 0.13 and that in the right breast was 0.49 ± 0.13, *p* >  0.05.

Sensitivity and Specificity of Monofrequency Electrical Impedance Mammography (EIM).

The distribution of the BIRADS diagnosis with MEIK electroimpedance mammography is shown in Table 4 and was as follows: BIRADS 1 (*n* = 211, 17.58%), BIRADS 2 (*n* = 765, 63.75%), BIRADS 3 (*n* = 173, 14.41%), BIRADS 4 (*n* = 46, 3.83%) and BIRADS 5 (n = 4, 0.33%). The distribution of the ACR BIRADS diagnosis by conventional mammography was as follows: BIRADS 0 (*n* = 51, 4.24%), BIRADS 1 (*n* = 135, 11.25%), BIRADS 2 (*n* = 505, 42.08%), BIRADS 3 (*n* = 74, 6.16%), BIRADS 4 (*n* = 20, 1.66%) and BIRADS 5 (*n* = 3, 25%). The distribution of the ACR BIRADS diagnosis by Doppler ultrasound was as follows: BIRADS 1 (*n* = 27, 15.16%), BIRADS 2 (*n* = 114, 64.0%), BIRADS 3 (n = 27, 15.16%), BIRADS 4 (*n* = 9, 5.05%) and BIRADS 5 (n = 1, 0.56%) (Table 4).

**Table 4:** Correlation of BIRADS Electroimpedance Mammography EIM and BIRADS Mammography

BI-EIM	BI-RADS Mammography						
	0	1	2	3	4	5	N
1	2	52	74	13	2	0	143
2	34	69	342	36	3	0	484
3	9	10	75	21	3	0	118
4	6	4	14	4	9	2	39
5	0	0	0	0	3	1	4
	51	135	505	74	20	3	788

The diagnosis of the certainty of breast cancer together with the sensitivity, specificity and positive and negative predictive values of EIM were determined as follows: 85% sensitivity [true positives/true positives + false negatives] × 100; 96% specificity [true negatives/true negatives + false positives] × 100; 12% positive predictive value [true positives/true positives + false positives] × 100; 99% negative predictive value (NPV) [true negatives/true negatives + false negatives] × 100 (Table 5).

**Table 5:** Sensitivity, Specificity and Positive and Negative Predictive Values of Electroimpedance Mammography EIM

BI-RADS EIM	Benign	Malignant
4, 5	44	6
1,2,3	1149	1
Sensitivity	85%	
Specificity	96%	
Positive predictive value (PPV)	12%	
Negative predictive value (NPV)	99%	

In total, 1200 mammography electroimpedance studies were performed and compared with other imaging modalities (Doppler ultrasound and conventional mammography), which were interpreted by certified radiologists. The cases with suspected malignancy (BIRADS 4 and 5 of the ACR) were biopsied and histologically reported (*n* = 13). True-positive (*n* = 6) and false-negative (*n* = 1) examinations in all patients were based on biopsy-proven cancer (ductal carcinoma-in situ and invasive cancer).

Sensitivity was defined as the percentage of cancers detected (with a biopsy and ultrasound or mammography) among all cancers detected with any modality: TP/(TP + FN), 6/(6 + 1) = 0.85 (7 malignant biopsies), where TP is true-positive and FN is false-negative. Specificity was defined as the percentage of normal results from the examination (with a specific imaging modality) of any area of the breast where cancer was not detected with any modality: TN/(TN + FP), 1148/(1149 + 44) = 0.96. Table 6 shows the 44 false positives, 6 biopsies were performed that were benign, and the other 38 were analysed by mammography and Doppler ultrasound, resulting in no suspicion of malignancy (Table 6).

**Table 6:**
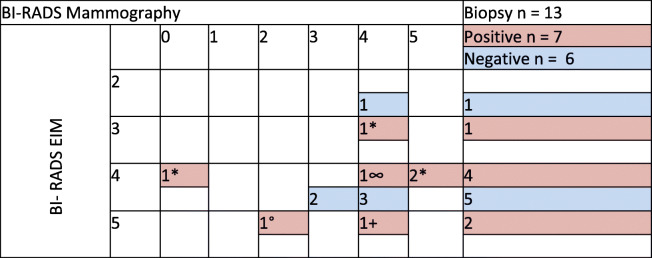
Breast Imaging Reporting and Data System for EIM and Mammography, Correlation with Biopsy and Histopathological Diagnosis

BI-RADS: Breast Imaging Reporting and Data System

*IDC: infiltrating ductal carcinoma, +ILC: infiltrating lobular carcinoma, °DCIS: ductal carcinoma in situ; ∞CC: canaliculus carcinoma

Distribution of mammary gland structure and density types from the perspective of EIM execution according to the ACR classification.

The mammary density from the EIM classification according to the ACR classification was as follows: amorphous *n* = 63 (5.25%), mixed with the predominance of the amorphous component *n* = 219 (18.25%), mixed *n* = 775 (64.5%), mixed with the predominance of the ductal component, high density of the ductal component *n* = 98 (8.16%), and extremely high density of the ductal component *n* = 44 (3.6%). Table 7 summarizes the results of the mammary gland density assessment from the perspective of electrical impedance mammography with regard to the electric conductivity index.

**Table 7:** Distribution of Mammary Gland Structure and Density Types

	EIM CLASSIFICATION – Electric Conductivity	n (%)	ACR
Type Ia	Amorphous IC, > 0.66	64 (5.33)	Predominantly fat, parenchyma below 25%
Type Ib	Mixed with the predominance of the amorphous component, 0.57–0.65	219 (18.25)	
Type II	Mixed, 0.30–0.56	775 (64.58)	Fat with some fibroglandular tissue, parenchyma between 25 and 50%
Type III	Mixed with the predominance of the ductal component, high density of the ductal component,0.22–0.29	98 (8.16)	Heterogeneously dense,parenchyma 50–75%
Type IV	Ductal, extremely high density of the ductal component, < 0.22	44 (3.66)	Extremely dense, parenchyma 75–100%

### Electric conductivity and body mass index

**Fig. 3:**
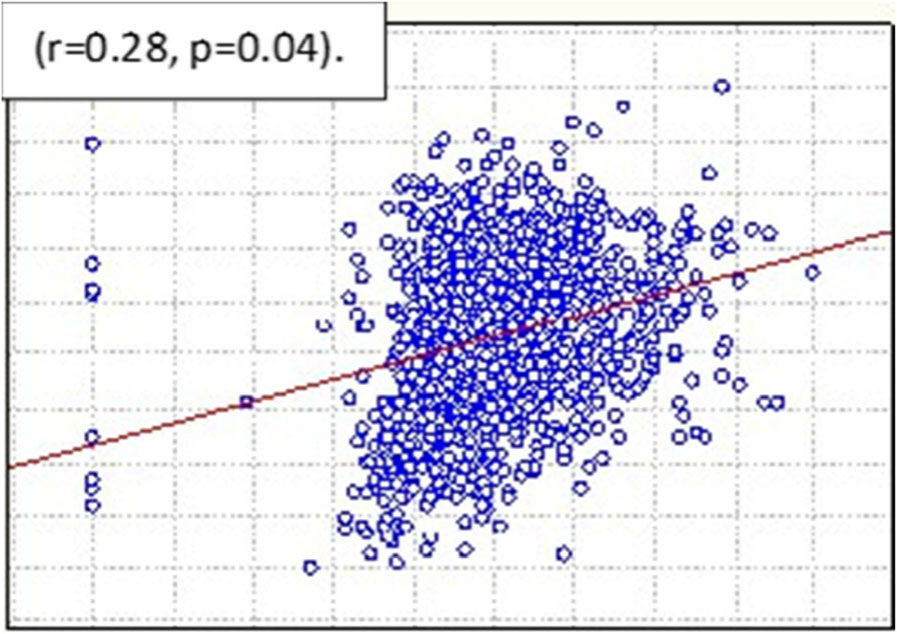
Correlation of electric conductivity and body mass index. *p* < 0.05 was considered significant

Electric conductivity was associated with body mass index, and we observed a statistically significant correlation (r = 0.28, *p* <  0.04) (Fig. 3). We used the chi-squared test to assess the interactions between percent density and body mass index (normal < 25 kg/m2 (*n* = 310), overweight 25–29.9 kg/m2 (*n* = 418) and obese ≥30 (*n* = 437)) (*p* = 0.04). The patients with a diagnosis of mammary carcinoma had a body mass index of 35.51 kg/m2.

**Fig. 4:**
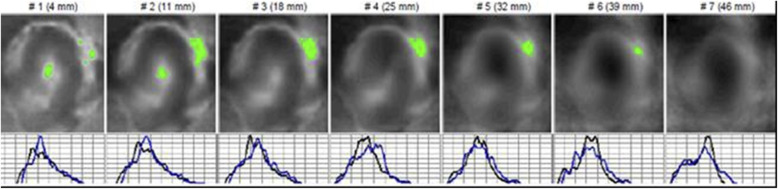
Electroimpedance Mammography. A series of 7 cuts of approximately 7 mm each. The left breast shows the following: mammary contour with extrusion in the radius of the 3 that displaces the mammary structure and generates distortion of the architecture. Mammary anatomy with changes marked; oval type with electroconductivity of 0.39, which indicates a lesion suspicious of malignancy since it is above 0.95

**Fig. 5:**
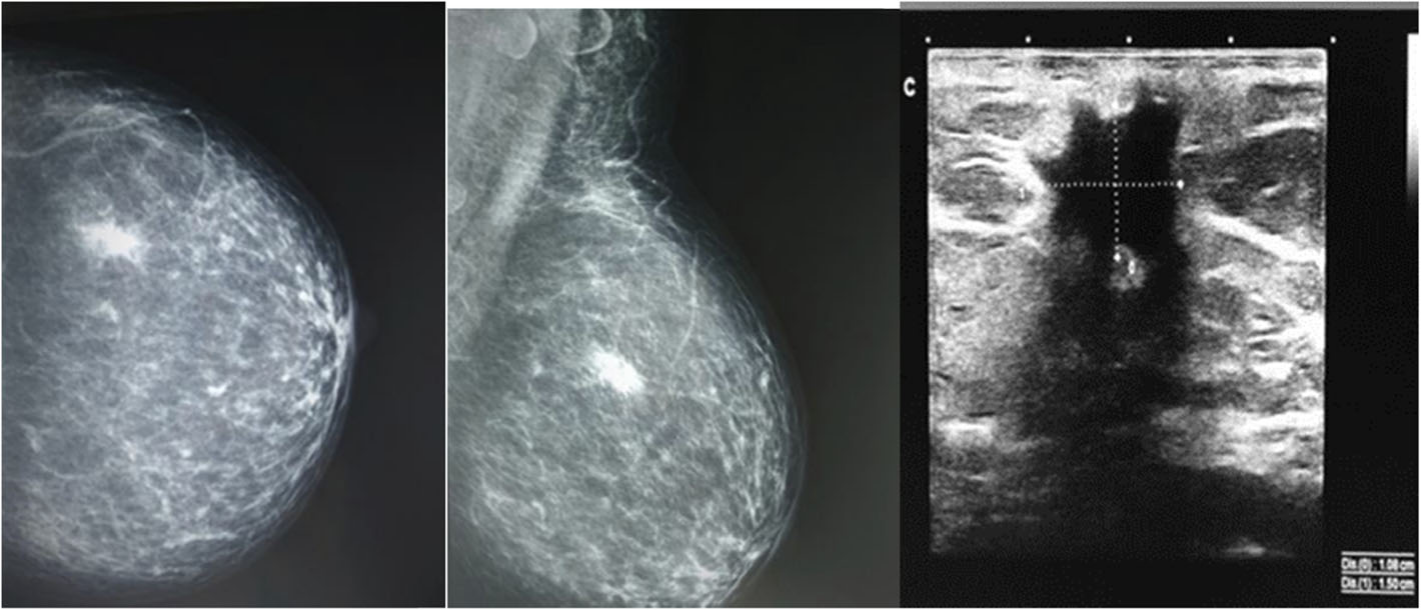
At 3 o’clock next to the areola, a focus is visualized, highlighted by the arrow. **a**, **b** X-ray: composition of tissue type B, a lesion 10 mm in size with radiant contour in the upper-outer segment. **c** US: A lesion of an irregular shape, partially angulated, undefined, hypoechoic margin, with a major axis not parallel to the plane of the skin, central vascularity to Doppler, and posterior acoustic shadow, in a radius of 3 to 5 cm of the nipple, with dimensions of 15x9x10 mm, 6 mm in depth. Category 5 of BI-RADS suggestive of malignancy merits histopathological correlation

The case of a 63-year-old asymptomatic patient who underwent exploration without positive palpation is described below. On admission, a mammography study was performed by electroimpedance (Fig. 4), followed by bilateral mastography and ultrasound (Fig. 5). A trucut biopsy was performed with a histopathological diagnosis of ductal carcinoma.

## Discussion

This study investigated the electrical impedance properties of breast tissue and demonstrated the different characteristics of electrical impedance scanning (EIM) images in groups of women of different ages, from 25 to 70 years old. Fuchsjaeger MH et al. (2005) performed a prospective trial to discriminate benign lesions from malignant lesions with a classification of BI-RADS 4 by mammography by means of electroimpedance in comparison with ultrasound, focusing on the negative predictive value [19]. There are several authors who have analysed the benefits of different techniques for the early diagnosis of breast cancer [20].

Stojadinovic A et al. reported that 50 of 189 women in the sensitivity arm had verified cancers, 19 of whom had a positive electrical impedance scanning (EIS) test that resulted in a sensitivity of 38% (19/50). Of the 1361 women in the specificity arm, 67 had a positive EIS test that resulted in a specificity of 95% (1294/1361) [21]. In the present study, we demonstrated that monofrequency electrical impedance mammography has a sensitivity of 85% and a specificity of 96% in 1200 women between 25 and 70 years of age, confirming the findings of previous studies. Glickman et al. Reported the results of an independent test group with 87 carcinomas, 153 benign cases and 356 asymptomatic cases. Histology was only available in symptomatic cases. The sensitivity was 84% with a specificity of 52% [22]. Malich et al. examined 387 lesions with the initial setup and found an overall sensitivity of 79% and a specificity of 64% [23].

Fuchsjäger et al. found the same increased sensitivities for smaller cancer, and the increased sensitivity for small malignant lesions could indicate the potential of this method to increase the rate of the early detection of breast cancer with minimal economic costs and highly qualified staff time expenditures [24]. We included visible lesions by ultrasound that were located posteriorly to EIM in the suspected area. The high specificity is the result of a low number of false positives.

In 2002, Fuchsjaeger and colleagues further explored the adjunctive role of EIS in 121 patients with 128 BI-RADS 4 lesions identified on mammography. Specifically, the results of EIS were compared with those of ultrasound as a technique of further classifying benign lesions such that patients could be managed as having a BI-RADS 3 lesion with a recommended six-month follow-up instead of biopsy [19]. Therefore, in this setting, the most relative statistic is the NPV, which can be used to exclude patients from biopsy. Based on histopathology from a subsequent biopsy, there were 37 malignant lesions and 91 benign lesions. The NPV of EIS was 97.1%, and that of ultrasound was 92.0%. It is unclear whether this diagnostic performance would be adequate to defer biopsy.

With an NPV of 99% of EIM for BI-RADS category 4 breast lesions, a negative result for these lesions could be a firm indication to manage them as BI-RADS category 3 and refer patients to a short 6-month interval follow-up rather than performing a biopsy [25]. Negative cases were followed for at least 1 year without evidence of cancer.

In our study, the NPV was 99%; the cases that were diagnosed by EIM as BIRADS 4 (*n* = 44) were deselected by ultrasound (*n* = 38), and only 6 that were benign were biopsied. The PPV was very low at 0.12. We attribute this to the age of the population studied (which was 25 to 70 years old), the age difference between the groups (and the difference in sensitivity by age), and that the cases of asymmetric density increased the number of BIRADS 4 by EIM.

Many investigations have been conducted to establish an association between obesity and breast cancer [26, 27]. It is necessary to establish methods that allow us to select, in patients with high breast density, those with a high risk of breast cancer to undergo complementary studies and/or breast biopsies to diagnose cancers in the early stages. Obesity and high breast density are common risk factors for breast cancer [28]. In our study, 7 patients with mammary carcinoma had a body mass index of 30.64. Shien Y et al. found a significant correlation between the percentages of mammary density and body mass index [29]. We analysed the distribution of conductivity and mammary density and found a relationship with body mass index. We found that 40% of postmenopausal women have a high body mass index. The average conductivity was 0.62 ± 0.04 in category 1 of the ACR (predominantly fat, parenchyma below 25%) vs. 0.17 ± 0.03 in category 4 (extremely dense, parenchyma 75–100%).

Chiu et al. reported that dense breast tissue was significantly associated with breast cancer incidence [relative risk (RR) = 1.57 (1.18–1.67)] and with breast cancer mortality [RR = 1.91 (1.26–2.91)] after adjusting for other risk factors and found that dense tissue was significantly associated with increased mortality from breast cancer [30].

Electroimpedance contributes to an evaluation with greater sensitivity in dense breast tissue and oestrogen use in postmenopausal women [31]. Recently, an important novel role has been investigated for EIM as a primary screening technique in younger women (less than 40 years) at average risk of developing breast cancer [32]. Currently, there are no specific screening recommendations other than breast self-examination in this population, in part due to decreased sensitivity of mammograms in imaging dense breasts, which are common in younger populations. EIM is based on the difference in electrical conductivity in benign versus malignant tissue and is not impacted by breast density [33]. The method may also be used as a screening method by young women, by women with dense breasts and by women with a high risk of carcinogenesis.

On the other hand, women < 40 years of age are not screened, and the majority of these women have dense breasts, as observed in our study. In addition to biological causal effects, dense breasts also have a masking effect that leads to a high rate of interval cancers due to a lower sensitivity, particularly in young women [34].

These results suggest that breast electroimpedance can be used as a complementary examination to conventional mammography and ultrasonography in women with dense breasts. The sensitivity of conventional mammography in publications worldwide varies between 40 and 98%. Lower sensitivity is mainly attributed to the dense breasts of younger women and the use of hormonal substitute therapy.

These are some advantages of electroimpedance mammography: non-invasive examination, the possibility of a frequent revision of the diagnosis, non-harmful examination, a relatively economic and inexpensive device, the non-expensive operation of the device, and the high sensitivity, especially in young women.

This study has a number of limitations, including the low prevalence of cancer in this population. More large-scale, long-term follow-up studies are needed in the populations of intended use. In addition, 6-month follow-up and surveillance of cases with BIRADS 4 were performed to determine whether they developed breast carcinoma.

## Conclusions

Our study showed that the sensitivity and specificity of monofrequency electrical impedance mammography (EIM) were 85 and 96%, respectively. This non-radiation method may also be used as a screening method for young women with dense breasts and a high risk of carcinogenesis.

These findings may support further clinical study and lead to EIM being reclassified from an experimental modality to an acceptable adjunct modality for the early detection of breast cancer.

## Data Availability

The data sets generated and / or analyzed during the current study are not publicly available due to [consent to publication of identifying images or other personal or clinical details of participants who compromise anonymity] but are available at the corresponding author at a reasonable request.

## Abbreviations

EIM: Monofrequency Electrical Impedance Mammography
US: Ultrasound
IC: Electrical Conductivity Index
PPV: Positive Predictive Value
NPV: Negative Predictive Value
ACR: American College of Radiology
BI-RADS: Breast Imaging Reporting and Data System
IDC: Infiltrating Ductal Carcinoma
ILC: Infiltrating Lobular Carcinoma
DCIS: Ductal Carcinoma in situ
CC: Canaliculus Carcinoma
BMI: Body Mass Index
